# *Actephilaalanbakeri* (Phyllanthaceae): a new nickel hyperaccumulating plant species from localised ultramafic outcrops in Sabah (Malaysia)

**DOI:** 10.1186/s40529-016-0122-1

**Published:** 2016-02-09

**Authors:** Antony van der Ent, Max van Balgooy, Peter van Welzen

**Affiliations:** 1 Laboratoire Sols et Environnement UMR 1120 UL - INRA, Université de Lorraine, 54000 Nancy, France; 2 Centre for Mined Land Rehabilitation, Sustainable Minerals Institute, The University of Queensland, St Lucia, Brisbane, QLD 4072 Australia; 3 Naturalis Biodiversity Center, Botany, Leiden, 2300 RA The Netherlands; 4 Institute of Biology, Leiden University, Leiden, 2300 RA The Netherlands

**Keywords:** *Actephila*, Malaysia, Phyllanthaceae, Sabah, Ultramafic

## Abstract

The Malaysian state of Sabah on the Island of Borneo is emerging as a hotspot for nickel hyperaccumulator species with at least 25 such species discovered to date. New discoveries of the hyperaccumulation trait in described taxa, as well as taxonomical novelties that are nickel hyperaccumulators, continue to be made. Here we describe a new nickel hyperaccumulating species of *Actephila* (Phyllanthaceae) originating from two known populations on ultramafic soils in Sabah. The most characteristic feature of *Actephila alanbakeri* are its knobbly fruits, but other diagnostic morphological characters are discussed and information about its ecology and rhizosphere and plant tissue chemistry is provided. This new species is one of the strongest known nickel hyperaccumulator plants in Southeast Asia with up to 14,700 μg g^−1^ (1.47 %) nickel in its leaves. The occurrences of *Actephila alanbakeri* on just two sites, both of which lie outside protected areas and are disturbed by recurring forest fires, combined with the small total numbers of individuals, render this species Endangered (EN) on the basis of IUCN Red List Criteria.

## Background

Ultramafic soils represent a category of substrates derived from ultramafic bedrock and are sparsely distributed around the world (Brooks 1987). These soils are known for relatively high concentrations of potentially phytotoxic trace elements, mainly nickel (Ni), as well as major cation imbalance and nutrient-deficiencies (Proctor 2003). The ultramafic soils of the Malaysian state of Sabah on Borneo Island are extensive, occupying an area of about 3500 km^2^ (Proctor et al. 1988) and are renowned for their high species richness with at least 2500 different plant species known to date (Van der Ent et al. 2015a). Some plants restricted to ultramafic soils have evolved ecophysiological mechanisms to tolerate and accumulate Ni, and are termed Ni hyperaccumulators when having in excess of 1000 ug g^−1^ Ni in their leaves (Jaffré et al. 1976; Reeves 1992, 2003; Van der Ent et al. 2013a). This phenomenon is rare and known in approximately 450 species worldwide in many different plant families (Reeves 2003; Van der Ent et al. 2013a). Nickel hyperaccumulators can be categorized into ‘obligate’ and ‘facultative’ hyperaccumulators (Pollard et al. 2014). Obligate hyperaccumulators are exclusively found on ultramafic soil and all populations of the particular species are hyperaccumulators. However, species that are ‘facultative’ hyperaccumulators have populations on ultramafic soils that are Ni hyperaccumulators, and populations on other soils that are not (Van der Ent et al. 2013a; Pollard et al. 2014). Hyperaccumulation is hypothesized to have evolved to interfere with other competing plant species (‘elemental allelopathy’) or to protect against insect herbivores (‘elemental herbivory defense’), although a variety of other explanations have also been proposed (Boyd and Martens 1998; Boyd and Jaffré 2001; Boyd 2012). Recent research activities have revealed the existence of at least 25 different Ni hyperaccumulator species in Sabah, making it a global hotspot for such plants (Van der Ent et al. 2013c, 2015b, c). The majority of Ni hyperaccumulating species in Sabah appear to be restricted to a single site or a few ultramafic outcrops and are hence rare and possibly threatened (Van der Ent et al. 2015b).

*Actephila* Blume is a plant genus in the tribe Poranthereae of the family Phyllanthaceae (Hoffmann et al. 2006). The genus comprises approximately 40 species ranging from Southeast Asia to the Pacific Islands (Govaerts et al. 2000). Typical for the genus are the generally single, axillary flowers with the petals smaller than the sepals. In the staminate flowers disc lobes are present and the stamens are free (or basally united) and centred around a three-fid pistillode. The pistillate flowers have a ring-like disc and a three-locular ovary with two ovules per locule. During fieldwork undertaken in 2011–2013 a new species of *Actephila* was collected on a hill near Nalumad, at the boundary of Kinabalu Park in Sabah, Malaysia. Later we also found a herbarium specimen that matched this taxon that was collected from Malawali Island, also in Sabah.

## Methods

Specimens were examined in Leiden, The Netherlands, from herbarium material sent from the Sabah Parks Herbarium (SNP) in Malaysia. The first author undertook fieldwork in Malaysia during 2011–2013. Soil samples (n = 3) were collected near the roots of the new *Actephila* species and air-dried at room temperature for 3 weeks before laboratory analyses. The soil pH was measured in a 1:2.5 soil:water mix. Soil sub-samples were extracted with DTPA (for phytoavailable trace elements, such as Ni) and with silverthiorea (for exchangeable cations, including calcium, magnesium, potassium and sodium) solutions (Becquer et al. 1995; Dohrmann 2006). Soil sub-samples were also digested with concentrated nitric (70 %) and hydrochloric (37 %) acid in a specialised microwave. Plant part samples (roots, twigs, bark, phloem, wood, leaves) collected from mature plants of the new *Actephila* were immediately washed with demineralised water, oven-dried at 70 °C and then digested with concentrated nitric acid (70 %) and hydrogen peroxide (30 %) in a specialised microwave. The soil and plant part sample extracts were finally measured with ICP-AES (Varian Vista Pro II) for Ni, Co, Mn, Fe, Mg, Ca, Na, K and P. The ICP-AES instrument was calibrated using a 6-point multi-element standard (Ni, Co, Mn, Fe, Mg, Ca, Na, K, P) prepared in each extraction solution. The laboratory work was undertaken at The University of Queensland, Australia.

## Results

### Taxonomic treatment

*Actephila alanbakeri* Welzen and Ent, sp. nov. —TYPE: MALAYSIA. Sabah. Near Kinabalu Park, Nalumad, Antony Van der Ent et al. SNP 38,539 (holo SNP; iso L). Paratype: SAN 145750, Malawali Island Kudat District, Sabah, Malaysia, John B. Sugau and Dauni Seligi (L, SAN).

This species differs in the knobbly fruits from all other species of *Actephila*, which have smooth fruits. The leaves dry to a distinct yellowish green.

Shrub, 3 m high, monoecious; flowering branchlets angular when dry, striate, c. 2 mm diam., greenish. *Indumentum* of simple hairs, most parts glabrous. *Stipules* triangular, 1.4–1.5 by 1–1.2 mm, persistent, stiff, brown when dry, base triangular in transverse section, margins slightly erose, glabrous except for basal row of up to 0.4 mm long papillae-like hairs. *Leaves* alternate, simple; petiole 2.5–8 mm long, deeply grooved above, smooth, glabrous; blade elliptic, 5.1–15.5 by 1–3.3 cm, 4.7–5.1 times as long as wide, symmetric, coriaceous, base slightly rounded, margins entire, revolute, apex acute, tip blunt, glabrous, drying yellowish green, duller and lighter below, venation pinnate, slightly raised above, raised below, secondary nerves 13–17 pairs, arching and closed near margin, higher order veins reticulate. *Inflorescences* axillary fascicles, containing a single flower in the type; bracts as stipules, slightly smaller, outer one c. 1 by 1 mm. *Staminate flower* c. 3 mm diam.; pedicel c. 3 mm long, somewhat angular when dry, pink and round when fresh, glabrous; sepals 5, ovate, c. 2 by 1 mm, free, apex acute, central part thicker, margins more membranous, pinkish red, outside hairy, glabrous inside; petals five, alternisepalous, shorter than sepals, spade-like, c. 1 by 1 mm, membranous, pinkish white, glabrous; disc thick, ring-like to partly free lobes, lobes episepalous, emarginate, whitish; stamens 5, epipetalous, glabrous, filaments c. 1.2 mm long, apically bent outwards, slightly tapering towards the apex, anthers basifixed, c. 0.4 by 0.5 mm, 2-thecate, thecae separate from each other, opening with apical transverse slit, pollen yellow; pistillode c. 1 mm long, tri-partite, hairy, pinkish red. *Pistillate flowers* not seen. *Fruits* 5- or 3-locular, lobes around seeds, knobbly all over. Similar fruits are known in Euphorbiaceae s.l. e.g., *Dimorphocalyx*, but not in Phyllanthaceae, formerly included in Euphorbiaceae (Van Balgooy 1997).

### Etymology

The specific epithet “*alanbakeri*” honours Professor Alan J. M. Baker, who is a leading pioneer in the discovery and global research on hyperaccumulator plants and in developing phytomining technology. Research by Professor Baker and his international collaborators since the late 1970s led to the discovery of most of the hyperaccumulator plants known to science today.

### Phenology

As far as known this species flowers and fruits all year round.

### Distribution and habitat

The species appears restricted to a ridge (‘Lompoyou Hill’) near the villages of Nalumad and Pahu, and from Malawali Island, both in Sabah, Malaysia. At the site near Nalumad, the hill (400 masl) has been burnt at least once as a result of an uncontrolled forest fire in the late 2000s. Prior to burning, the site had already been disturbed by logging. At present the site has a short and open scrub community (dominated by shrubs 1–3 m tall) with pioneer species such as *Macaranga kinabaluensis* Airy Shaw (Euphorbiaceae). In this habitat type several other Ni hyperaccumulator plants occur, including *Phyllanthus balgooyi* Petra Hoffm. and A. J. M. Baker and *P.* cf. *securinegioides* Merr. (Phyllanthaceae), *Mischocarpus sundaicus* Blume (Sapindaceae), *Rinorea javanica* Kuntze (Violaceae), *Psychotria sarmentosa* Blume (Rubiaceae) and *Xylosma luzonensis* Clos (Salicaceae). The local conditions are pronouncedly xeric, and the soils are shallow and heavily eroded, revealing the underlying saprolite. *Actephila alanbakeri* occurs sporadically in this scrub with scattered individuals mainly on the ridges. Little information is available for the collection from Malawali Island, except that the *Actephila* grew on ultramafic soil at 79 masl in forest.

### Soil chemistry

The soils of the habitat near Nalumad in which *Actephila alanbakeri* occurs are heavily eroded due to the lack of vegetation after forest fires. Intensive weathering has since exposed the saprolite layer and associated bedrock (which consists of strongly serpentinised peridotite). The soils are mainly shallow Magnesic Ferralsols characterised by their very high iron and magnesium content, which is typical for ultramafic soils (Brooks 1987). The analyses of soil samples (Table 1) show that the soil pH is near neutral (pH 6.7), and chemically the soil is extreme in containing high concentrations of total and exchangeable magnesium (Mg), and high nickel (Ni) and manganese (Mn). The soil major nutrient (Ca, K, P) concentrations are low, another characteristic of ultramafic soils.

**Table 1:** Soil chemistry in the rooting zone of *Actephila alanbakeri* (the number of samples is three with ranges and means provided)

Parameter	Range
pH	6.4–7.0 [*6.7*]
Co (total) μg g^−1^	122–670 [*369*]
Mg (exch.) μg g^−1^	1710–2830 [*2250*]
K (exch.) μg g^−1^	102–228 [*153*]
P (total) μg g^−1^	20–182 [*76*]
Co (DTPA) μg g^−1^	13–45 [*25*]
Cr (total) μg g^−1^	1930–4480 [*3240*]
Ca (total) μg g^−1^	1150–6990 [*3160*]
Ca (exch.) μg g^−1^	1810–4500 [*2920*]
Fe (total) mg g^−1^	104–177 [*142*]
Mg (total mg g^−1^	24–41 [*31*]
Mn (total) mg g^−1^	2.7–6.1 [*3.9*]
Ni (total) μg g^−1^	1000–2330 [*1790*]
Ni (DTPA) μg g^−1^	171–226 [*199*]

### Plant foliar chemistry

When the plant was first found, field-testing with dimethylglyoxime (DMG), a nickel-specific colorimetric reagent, revealed its Ni hyperaccumulating trait. Subsequent laboratory analysis of leaf samples confirmed this (Table 2), and showed that foliar Ni concentrations are up to 14,700 μg g^−1^ (or 1.47 %) with a mean concentration of 5800 μg g^−1^ Ni. These are amongst the highest foliar Ni concentrations recorded globally, and this species falls into a category of hyperaccumulator plants termed ‘hypernickelophores’ (Jaffré and Schmid 1974), which are plants with >1 % foliar Ni numbering only approximately 150 species globally. The foliar Ni concentrations in *Actephila alanbakeri* are in Sabah only exceeded by *Phyllanthus* cf. *securinegioides, Glochidion* sp. ‘bambangan’ (Phyllanthaceae)*, Psychotria sarmentosa* (Rubiaceae), and *Rinorea bengalensis* (Violaceae) with (23,300 μg g^−1^, 16,700 μg g^−1^, 24,200 μg g^−1^ and 12,800 μg g^−1^, respectively (Van der Ent et al. 2015b). Apart from the high Ni concentrations, foliar Ca and K concentrations are also unusually high, despite the low concentrations of these elements in the soil. Nickel concentrations in the different plant parts were also determined, and are as follows: twigs 591 μg g^−1^ (mean value), phloem tissue 2490 μg g^−1^, root 970 μg g^−1^, bark 1960 μg g^−1^ and wood 270 μg g^−1^ [new data augmented by data from (Van der Ent et al. 2015c), note that the *Actephila alanbakeri* was designated as *Cleistanthus* sp. nov. in that publication]. Despite the high foliar Ni concentrations, at levels presumably toxic to most insects, abundant herbivory damage on the leaves of *Actephila alanbakeri* does not support the hypothesis that Ni hyperaccumulation affords protection, unless the herbivory damage is inflicted by specialised “high Ni-tolerant insects” (Boyd 2009).

**Table 2:** Plant part chemistry of *Actephila alanbakeri* (number of samples indicated, for leaves and twigs ranges and means are provided)

Plant part	n	Al μg g^−1^	Ca μg g^−1^	Co μg g^−1^	Fe μg g^−1^	K μg g^−1^	Mg μg g^−1^	Mn μg g^−1^	Ni μg g^−1^	P μg g^−1^	S μg g^−1^	Zn μg g^−1^
Leaves	11	12–58 [*31*]	1520–10,530 [*6430*]	13–95 [*43*]	16–101 [*63*]	8780–22,380 [*14,650*]	1130–6500 [*3350*]	52–933 [*361*]	280–14,700 [*5760*]	303–1440 [*755*]	609–2770 [*1780*]	21–227 [*113*]
Twigs	4	8.4–76 [*34*]	1280–4760 [*2980*]	13–32 [*22*]	8–12 [*10*]	6760–9240 [*7670*]	12,020–3010 [*2110*]	67–267 [*133*]	386–1700 [*868*]	748–1540 [*1010*]	409–988 [*719*]	23–76 [*48*]
Wood	1	5.8	202	8.9	14	1670	522	38	270	267	293	13
Phloem	1	30	2090	13	46	12,790	4250	351	2490	285	843	119
Bark	1	28	6770	15	51	8080	2920	193	1960	387	964	353
Root	1	64	310	8.4	297	3900	1260	26	970	351	214	43

### Phylogenetic occurrence of Ni hyperaccumulation

**Fig. 1:**
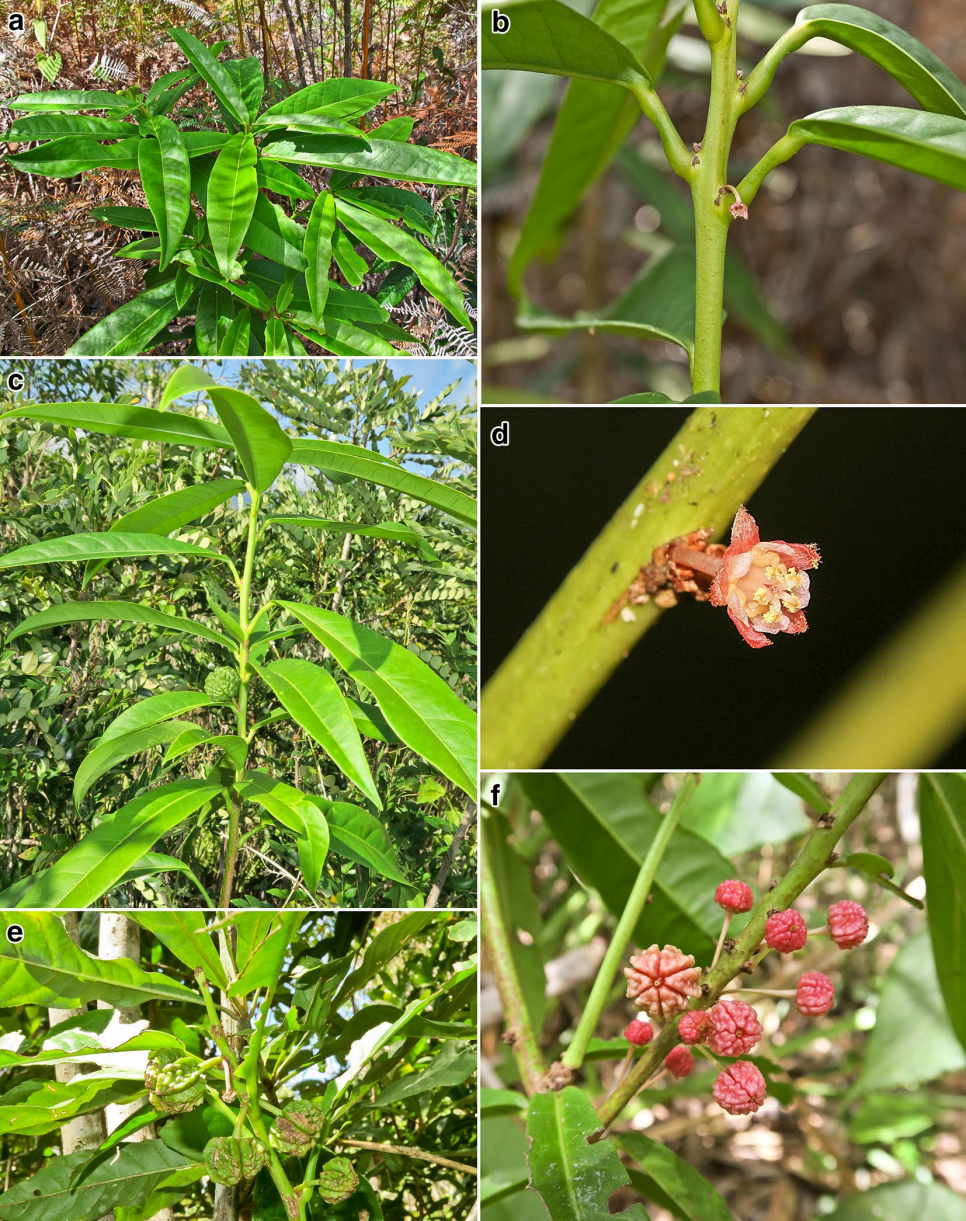
*Actephila* *alanbakeri* in the native habitat (**a**), habit of the plant with inflorescences (**b**), habitat of the plant with infructescence (**C**), close-up of inflorescence (**d**), ripe infructescences (**e**), closeup of young infructescences (**f**). Photos by Yabainus Juhalin, Sukaibin Sumail and Antony van der Ent

The main families in which Ni hyperaccumulation has been recorded in Sabah are the Phyllanthaceae, Rubiaceae, Salicaceae and Violaceae. This conforms to the global trends for Ni hyperaccumulator phylogenetic lineages, with these families also being the most important in other hotspots for Ni hyperaccumulator plants, such as in New Caledonia and Cuba (Reeves 2003). These families, except the Rubiaceae, are all in the order Malpighiales (Angiosperm Clade Rosids). The large family Phyllanthaceae (over 2000 species in 60 genera) has its centre of diversity in the Malesia and Australasian regions (Govaerts et al. 2000; Kawakita 2010). *Actephila alanbakeri* is the first record of a Ni hyperaccumulator in the genus *Actephila* (Fig. 1).

### Conservation status

The habitats of *Actephila alanbakeri* are outside any protected areas, on a hill close to Kinabalu Park, and on Malawali Island. The restriction of this species to just two populations, the small area of occupancy (<10 km^2^), and the small number of individuals (no more than 100 individuals have been observed to date, although no information is available about the population-dynamics and hence trends in population size) means that this species is sensitive to disturbances which could ultimately lead to its extinction. This is a real possibility because *Actephila alanbakeri* occurs in patches of remaining scrub in an area devastated by recurring forest fires. Therefore, the species can be classified as Endangered (EN) on the basis of IUCN Red List Criteria (Version 3.1: IUCN 2001).

### Outlook and further research

**Fig. 2:**
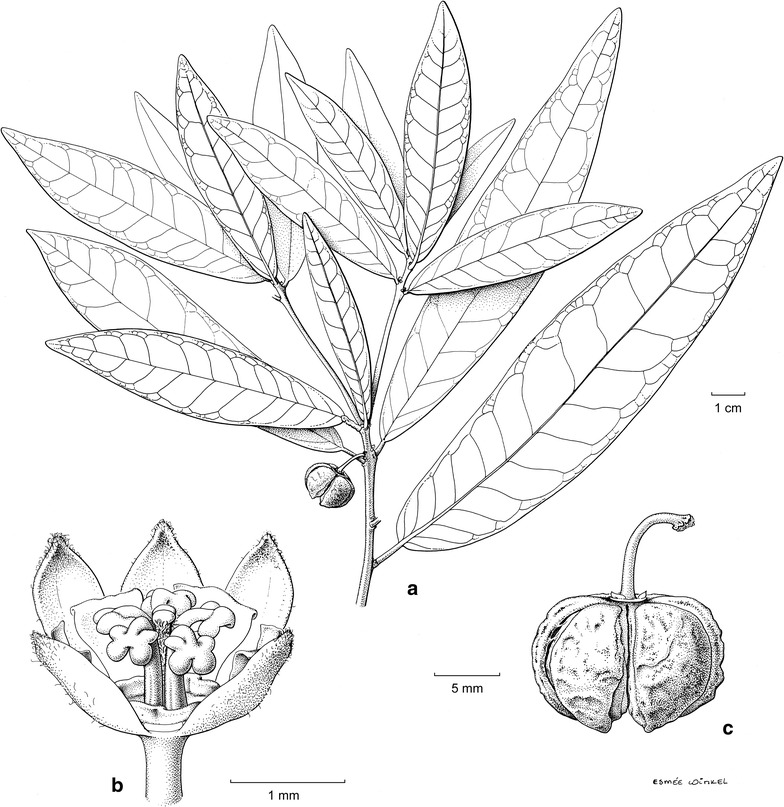
*Actephila alanbakeri* Welzen & Ent—**a** Habit; **b** staminate flower, front petal removed; **c** dehiscing fruit [**a**, **c**: SAN (John B.S. and Dauni S.) 145,750; **b**: SNP 38,539].—drawing: Esmée Winkel, 2015

With only a small portion of the ultramafic flora of Sabah screened for Ni hyperaccumulation, it is expected that more Ni hyperaccumulators will be discovered in the near future, especially in taxa from the order Malpighiales. Such screening also support strategies aimed at the preservation of hyperaccumulator taxa, many of which are rare, which is essential if they are to be utilised in any future Ni phytomining operations, aimed at growing ‘metal crops’ at agricultural scale to harvest Ni bio-ore (Van der Ent et al. 2013b, d). Sabah Parks has recently established the ‘Hyperaccumulator Botanical Garden’ at Monggis sub-station of Kinabalu Park. This facility is aimed at supporting ex situ conservation of rare and threatened Ni hyperaccumulator species, and to enable research on these unusual plants. Nearly all known Ni hyperaccumulator species (>25 taxa) from Sabah, including *Actephila alanbakeri*, are currently cultivated on naturally occurring ultramafic soil at the Hyperaccumulator Botanical Garden. It is hoped that this facility will promote further scientific investigations to better understand the ways in which hyperaccumulator plants tolerate extreme Ni concentrations in their living shoots (Fig. 2).
